# Runnability: A Scoping Review

**DOI:** 10.3390/ijerph22010071

**Published:** 2025-01-07

**Authors:** Ashley D. Tegart, Nadine Schuurman, Stella R. Harden

**Affiliations:** Faculty of Environment, Simon Fraser University, 8888 University Drive, Burnaby, BC V5A 1S6, Canada; ashley_tegart@sfu.ca (A.D.T.); stella_harden@sfu.ca (S.R.H.)

**Keywords:** running, runnability, scoping review, health and information technology

## Abstract

Running outdoors is an increasingly popular form of physical activity and has been proven to substantially reduce the risk of major chronic illnesses such as cardiovascular disease. The topic of runnability has received considerable attention but with conflicting conclusions and remaining gaps. The physical environment and its features impact running experiences. Detecting features facilitating and deterring runners is crucial to promoting this physical activity and, therefore, overall health. A scoping review of current literature was conducted to identify environmental factors conducive to running. Online databases were used to identify all articles on runnability to date; a total of one hundred and two (n = 102) papers were selected as they identified environmental correlates preferred by runners. Findings include a preference for green spaces and connecting with nature, perceptions of higher safety away from traffic congestion and pollution, and routes with wide, smooth surfaces and high connectivity. Essentially, natural surroundings are substantially more desirable than urban settings. Studies have shown that even when a running route is within an urban environment, it is usually connected to or between green spaces.

## 1. Introduction

Recreational running has increased in popularity in recent years [1,2,3] as it is relatively accessible and evidenced to improve overall health and well-being [4,5,6,7,8,9]. The concept of runnability was first described in 2021 “as a quantification of the features of the built environment that facilitate the movement of runners” [10]; it incorporates all types of runners and environments conducive to running [11].

Although studies have linked increased running activity within or in proximity to green spaces [12,13,14], little analytic attention has been given to the patterns and preferences of outdoor runners [2,12,15,16,17]. Some studies have analyzed the topic of runnability through walkability and/ or cycling patterns or indices [10,18,19,20,21]; however, the correlates preferred by runners will differ from other physical activities. For example, increasingly, people choose walking or cycling, overtaking their car to work or the grocery store. However, for runners, commuting to work and other locations is less common. 

The goal of this research is to identify characteristics of the environment that encourage or inhibit runnability. This will be accomplished through a scoping review, a near-exhaustive process of “knowledge synthesis that addresses an exploratory research question aimed at mapping key concepts, types of evidence, and gaps in research” [22]. We will identify the environmental variables conducive to running in natural and built environments; this will be accomplished through a thorough search for articles pertaining to running and the environment, analysis of their evidence, and presentation of runnability findings.

### 1.1. Background

The first reference to runnability in academic literature was through the creation of the Rough Runnability Index (RRI), which quantified the features of the built environment facilitating or hindering runnability [10]. This was followed by a cross-sectional survey that identified the preferences of recreational road runners, such as the type of paved surface [23], and an additional study researched correlates related to trail running [24]. An analysis of running through Strava data contributed to the foundational understanding of runnability characteristics such as green and blue spaces, urbanicity, and socioeconomic status [12]. Another approach was the comparison of outdoor runners to those who prefer indoor gyms, emphasizing the importance of experiencing nature while running; many runners experience a sense of relaxation while running in green spaces, while others believe it contributes to a greater quality of life [25,26,27]. Running in green spaces has also been associated with better performance and greater psychological well-being, especially for men [28,29]. While studying the importance of inclusivity while running, it was discovered that social events such as Parkrun encouraged participation predominantly for women and those who have less experience running—especially in green spaces [30,31]. Green spaces have been evidenced to promote physical activity, especially for women after life transitions such as childbirth [32]. In addition to the physical and aesthetically pleasing characteristics of the natural and built environment that affect runners’ performance, researchers noted detriments such as pollutants and trail safety concerns [33,34]. Our research team determined the need for a scoping review to capture all environmental correlates related to runnability.

### 1.2. Qualitative, Quantitative, and Mixed Method Approaches

One of the fundamental divides in runnability research is through methodological approaches and their applicability. Some studies focused on qualitative data, consulting runners through surveys, questionnaires, and diary interviews, while others preferred Big Data collection methods such as multi-source crowdsourced data analysis. One study argued that observational methods, along with questionnaires and diary entries, contributed to the main gaps in previous studies [35]; however, critics of quantitative studies noted approaches such as GIScience analysis alone may not produce generalizable findings. Furthermore, the difference in focal correlates contributes to runnability research gaps. For example, studies that observed changes in park visitation pre- and post-park developments may not reflect findings from studies focusing on the time of day runners visit a park or if participants preferred to run alone or with a group [36,37].

Researchers discovered that incorporating tracking data has helped determine known environmental correlates, which amplifies when the findings are combined with qualitative analysis. Spatial data by runners on social media and platforms such as Strava helps runners share routes and experiences. 

Harvesting Volunteered Geographic Information (VGI) acquired data provide runner route information such as popularity and frequency in use; however, increasingly, people want their data and privacy protected, and there has only been a single effort discussing the impact of VGI on runners discovered in this study [38]. 

### 1.3. GPS, GIS, and Big Data

Since the introduction of Big Data, crowdsourcing has been a popular method for data extraction for population representation. Many runners use apps to track their progress and routes. Information about runners and their preferences can be obtained from GPS trajectory running data from popular mobile exercise apps. Previous runnability research in our lab used GIScience methods to analyze Strava data focused on running routes, frequency, and intensity [10,35,39]. Other online public web data feeds in this study include MapMyRun.com [40,41,42] and the sports apps *Edooon* [43,44], *Adidas Runtastic* [45], *Tulipsport* [46], and *Keep* [47].

## 2. Materials and Methods

To fully understand runnability and its correlates, we reviewed current literature using the scoping review methodology. Scoping studies are an approach used for novel topics and to identify gaps in the literature. To date, a runnability scoping review has not previously been undertaken. The scoping review will provide a list of variables associated with runnability from the nascent runnability literature. The methodology relied on the framework stages developed by Arksey and O’Malley [48] and the PRISMA extension for the scoping reviews checklist [49]. A summary of the scoping review steps is found in Table 1 below: 

**Table 1:** Runnability Scoping Review Summary.

Stage 1 Identifying Research Question	Goal of Study	To determine the barriers and facilitators to running in the urban and natural environment
	Research Question	Which features of the built urban environment have been identified with running and runnability in the academic literature?
	Research Objectives	1. To determine the variables that make an environment runnable according to current literature; and 2. To determine geographical patterns and environmental features preferred by runners.
Stage 2 Identifying the Relevant Studies	Eligibility Criteria	The scoping review aimed to obtain all relevant literature through electronic databases and hand-searching references of key studies. Restricted to studies published in English and in online databases.
	Information Sources	The scoping review included all literature available within the eligibility criteria, including gray (unpublished) literature, conference papers, books, and book chapters.
	Variable Selection and Preparation	Variation in the variables was created to ensure relevant literature was not missed (Table 2 and Table 3).
	Database Selection	Three databases were selected to ensure a broad search with different foci: GeoBase [50], PubMed [51], and Web of Science Core [52].
	Search Protocols	For each database, two variables were searched using the Boolean operation “AND.”
Stage 3 Study Selection	Selection of Sources of Evidence	A total of 752 articles were selected due to their applicability and eligibility. A total of 284 articles remained after the removal of 468 duplicates (Table 4).
	Screening Process	102 articles remained after the committee meeting (Table 5 and Figure 1)
Stage 4 Charting the Data	Selection for charting	Articles were organized by search terms. Articles were charted in Excel: Author, name and date of the article, publishing journal, and abstract.
Stage 5 Summarizing and Reporting Results	Synthesizing Evidence	Each article was assessed, and themes were identified (Table 6).
	Presentation of Evidence	Presented in a narrative format, table, and diagram (Figure 2).

**Figure 1:**
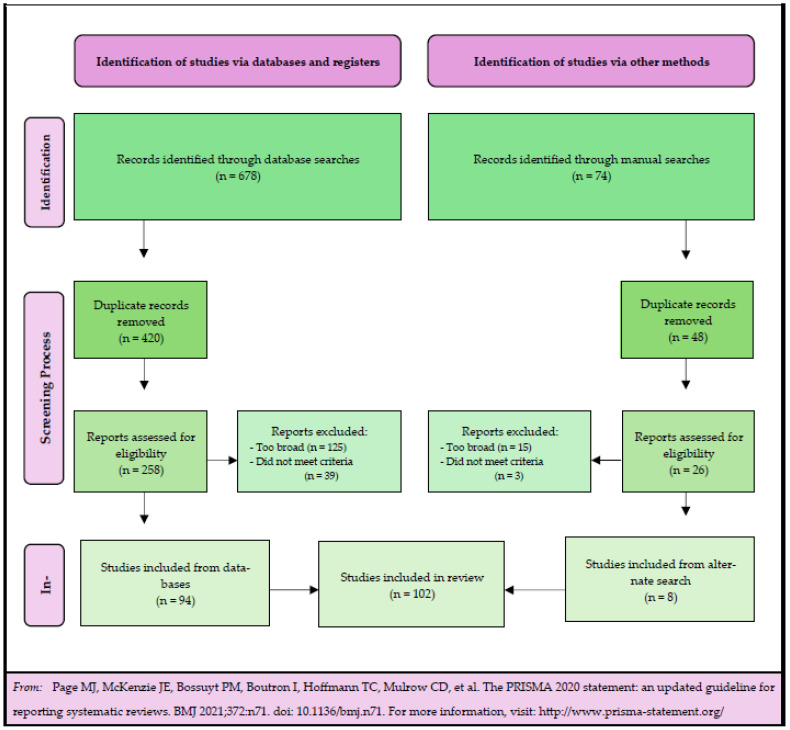
Runnability Scoping Review Flow Diagram.

**Figure 2:**
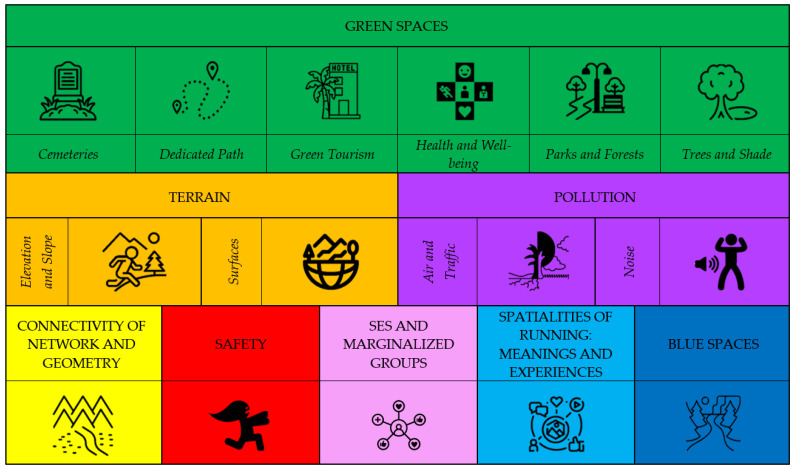
Runnability Key Themes.

**Table 2:** Runnability Scoping Review Search Terms.

Search Term 1			Search Term 2							
runnability	runner		road	safety	trees	bluespace	sidewalk	greenspace	vert	slope
running	jogging	jogger	trail	pollution	shade	blue space	park	green space	vertical	elevation

**Table 3:** Runnability Scoping Review Search Terms Exceptions.

Web of Science Adjustments Included [Physical Activity OR Exercise]			
running and road	running and slope	running and vertical	running and safety
running and trees	running and elevation		running and pollution

**Table 4:** Runnability Scoping Review Summary of Results.

Source	Web of Science		PubMed		GeoBase		Key Articles		TOTAL	
Articles	Total	Duplicate	Total	Duplicate	Total	Duplicate	Total	Duplicate	Total	Duplicate
	324	185	199	117	155	118	74	48	752	468
Total Articles sent to Committee for Review									(n = 284)	

**Table 5:** Deliberation and Results Summary.

Included Articles		Excluded Articles	
Articles unanimously agreed to include	(n = 81)	Articles unanimously agreed to exclude	(n = 157)
Total Articles for Deliberation (n = 46)			
Articles to include after deliberation	(n = 21)	Articles to exclude after deliberation	(n = 25)
Total articles included	(n =102)	Total articles excluded	(n = 178)

The runnability scoping review deliberation table summarizes articles included and excluded during the study selection stage of the scoping review process.

**Table 6:** Summary of Runnability Correlates in Current Literature.

Correlates 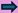	SAFETY	TERRAIN		CONNECTIVITY	GREEN EXERCISE						BLUE SPACES	SPATIALITIES	POLLUTION		SES
Author(s) 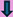		*Elevation and Slope*	*Surfaces*		*Cemeteries*	*Dedicated Path*	*Health and Well-being*	*Parks and Forests*	*Tourism*	*Trees and Shade*			*Air and Traffic*	*Noise*	
Adlakha et al. (2014) [40]							1	1							1
Alawadhi (2022) [53]	1		1					1		1					
[ … ]															
Total count:	37	21	41	28	1	27	38	63	12	17	32	32	31	14	37

This runnability scoping review was completed in five stages with meticulous transcription of data acquisition and methodology process (summarized in Table 1 above) with supervision and support from Simon Fraser University’s Geography, GIScience, and Maps research librarian. Using the scoping review process, we assigned one researcher for the article collection process (1 ‘initial researcher’) and two additional researchers for the review process (2 ‘reviewers’). The article applicability agreement process required three researchers (3 ‘committee researchers’), consisting of the ‘initial researcher’ and ‘research reviewers’. 

### 2.1. Identifying the Research Question

The goal of this study to determine the barriers and facilitators to running in the urban and natural environment was guided by the *research question*: Which features of the natural and built urban environment have been identified with running and runnability in the academic literature? 

To answer this research question, the methods addressed the following objectives:

(1) To determine the variables that make an environment runnable according to current literature; and
(2) To determine geographical patterns and environmental features preferred by runners.

### 2.2. Identifying the Relevant Studies

The primary interest of this review was to determine the environmental correlates of preference for outdoor runners. 

#### 2.2.1. Eligibility Criteria

As the study of runnability is new, the scoping review aimed to obtain all relevant literature, including books and chapters, conference papers, and both published and grey theses. A comprehensive search was conducted in electronic databases and with hand-searching references of key studies. The eligibility criteria included publishing restricted to the English language and online databases due to time constraints. There were no publishing period restrictions except those defined by the database. 

#### 2.2.2. Information Sources

To ensure a comprehensive search was completed, the scoping review included all literature available within the eligibility criteria. This included grey (unpublished) literature and hand-searching reference lists of relevant articles. 

#### 2.2.3. Variable Selection and Preparation

Variations in the correlates were created to ensure relevant literature was not missed. For example, as runnability is a new area of study, the search terms “running” and “runners” were included; “Greenspace” and “Green Space” (as examples) were also used due to variations in spelling. 

#### 2.2.4. Database Selection

Three databases were selected to ensure a broad search with different foci:

(1) GeoBase—provides international and interdisciplinary environmental searches with coverage from 1970 to the present [50].
(2) PubMed—primarily biomedicine and health-related sources; this database contains more than 36 million citations [51].
(3) Web of Science Core Collection—contains literature from the sciences and social sciences from 1900 to the present and 2.1 billion cited references [52].
(4) Google Scholar—a search engine that utilizes search robots to find and represent literature from other websites, journals, university repositories, books, theses, etc. Google Scholar was used for supplemental searches.

#### 2.2.5. Search Protocols

We discovered evidence to support key themes to be used as search terms to obtain the articles. For each database, two variables were searched using the Boolean operation “AND”: 

All results were then assessed for eligibility and applicability. Exceptions were made to include “physical activity OR exercise” using the Web of Science database for the following searches due to overwhelming irrelevant results: 

The search protocol was developed and executed by the research team with a strategy overseen by Simon Fraser University Geography, GIScience, and Maps librarian Sarah (Tong) Zhang. The initial researcher completed the scoping review study selection search. 

### 2.3. Study Selection

The runnability literature was compiled in Excel using database and variable pairings. The selection criteria required all literature to indicate a preference or motivation to facilitate or inhibit a runner to a particular environment or environmental characteristic. Duplicates were counted in each section.

#### 2.3.1. Selection of Sources of Evidence 

A total of 752 articles were selected due to their applicability and eligibility to runnability from the databases and hand-searching key articles. After the removal of 468 duplicates, a total of 284 articles remained for screening. The results were recorded in a table that identified all relevant results and their duplicates: 

#### 2.3.2. Screening Process

The screening process involved two research reviewers and did not include the initial researcher who completed the preliminary search for articles. The initial researcher explained the runnability criteria to the reviewers and provided a list of articles with the following information: author(s), publication date, publication origin (online journal information), and abstract. After the meeting was adjourned, the reviewers evaluated each article (n = 284) for its applicability without consultation and submitted it back to the initial researcher. All articles unanimously included (n = 81) were part of the scoping review. Any articles without a unanimous decision for their applicability (n = 46) were discussed in a following meeting for deliberation with all researchers.

#### 2.3.3. Disagreements

In total, there were 21 articles that were not initially unanimously agreed upon but were included in the scoping review after deliberation. Reasons for article removal included the ineligibility to explicitly meet criteria or the article’s focus being too broad. A summary of the deliberation process is summarized in Table 5. Figure 1 illustrates the major steps in the runnability scoping review.

The Runnability Scoping Review Flow Diagram (Figure 1) provides a summary of the scoping review process. It was adapted from the PRISMA 2020 flow sheet for new systematic reviews.

### 2.4. Charting the Data

The 102 agreed-upon articles were then assessed for key themes. One reviewer charted and inconsistencies were discussed among the committee researchers. Relevant information was extracted through a thorough assessment of articles, identifying patterns, and in-depth record-keeping.

### 2.5. Collating, Summarizing, and Reporting the Results 

Themes were obtained through the assessment of articles. The research committee used an iterative process to obtain the key runnability themes; as knowledge increased, the research committee redefined search terms and repeated analysis of articles to ensure a comprehensive understanding of the literature. Table 6 illustrates the major findings of the scoping review—the columns represent the referenced correlates in the current literature, and the rows represent the articles found in the runnability scoping review:

#### Synthesis of Results

All three members of the research committee completed data curation, formal analysis, and investigation to ensure the review results were unanimous. In the runnability scoping review, we have identified the key themes in relation to runnability. Figure 2 provides a summary of the results of the key correlates:

To answer the research question, “Which features of the natural and built urban environment have been identified with running and runnability in the academic literature?” The scoping review identified the correlates facilitating environment runnability by examining current literature. The evidence will be presented in a narrative format in the results section below.

## 3. Results

Most of the articles discussed many themes relating to runnability, and all themes were synthesized for analysis; our research committee selected (1) primary runnability theme in each paper at our discretion for organizational purposes only. A description and evidentiary research of each theme is summarized in Table 7:

While each article represented one or more correlates, results in the most preferable correlates differed greatly. Most research findings concluded Safety, proximity to Blue Spaces, and Connectivity of Networks and Geometry are the correlates most important to runners. The preference for Green Exercise (running in or near nature as opposed to an overwhelmingly built environment) was almost unanimous. 

Safety refers to protection from others or natural causes. Terrain breaks down the topic of Elevation and Slope (e.g., altitude and gradient, respectively), as well as preferences of different Surfaces of running paths. Connectivity of Network and Geometry discusses interruption of running flow from conflicts with others (e.g., traffic and other path users). 

Green Exercise is a broader category since most studies evidence green space and its attributes as correlates. Cemeteries, while presented with contrasting opinions, offer an explanation and alternate route in an ever-expanding urban environment. Dedicated Path offers insight into the accessibility and convenience of paths, as well as a preference for interconnected green spaces. Health and Well-being breaks down the multitude of reasons runners choose this form of physical activity from the psychological and physiological benefits. Parks and Forests are among the highest correlates to runnability as they encompass most of the other correlates in one area. A lesser-known Green Tourism explores the experiences of (typically) elite runners searching for added adventure in running segments. Finally, the topic of Trees and Shade discusses the implications of sun and wind protection from nature within and outside of green spaces. 

Blue Spaces or ‘bluespaces’ discusses the effects and associations of both man-made and natural water features and their impact on running speed and intensity. While more research is required, Spatialities of Running: Meanings and Experiences review runnability correlates beyond the physical environment: social needs, restorative benefits, and spiritual connections. Pollution from Air and Traffic and Noise has been shown to be a strong hindrance to runnability, sometimes preventing running from occurring. The results conclude with Socioeconomic Status (SES) and Marginalized Groups, an unintended yet not surprising discovery in runnability research; here, we discuss aesthetics, amenities, and overall runnability barriers rooted within prioritized development, harassment, and discrimination. 

Many of the results are complex, interlapping, and contradictory.

## 4. Discussion

### 4.1. Safety

Safety is a concern for many runners and an impediment to runnability [47,55,60,121]. Safety relates to the potential for injury and harm to one’s health. Running concerns include environmental factors, running experience, and risk of danger. Safety is further affected by time of day [38], seasonality [53,91], and interactions with others [1,2,10,15,23,23,35,43,65,72,111,121].

Environmental Factors—High heat [53], encounters with animals [23,24,35], slippery or unkept surfaces [23,24,35,74], and high vegetation cover [35,55] are impediments to runnability. Runners typically seek environments to protect from injury in meteorological conditions, such as running in the shade when it is hot [53] or running on pavement during the rainy season [21,91]; however, more experienced runners may view a dark and slippery forest to be an added challenge and therefore increase the runnability for some [91]. This is corroborated by the findings of Gordon et al. (2004), confirming safety rates as a higher priority, specifically for new runners [67].

Harassment and Interactions—Areas of perceived risk of sexual [54] and other forms of harassment [24,54,120], assault [24], encounters of racism [120], and low light [1,2,11,13,17,23,55,74,90,93,98] are generally avoided. Predominantly, women rate safety as a higher correlate to runnability [23,54]. Despite the benefits of inclusive running clubs [30], a less intimidating environment than a gym [32], adequate lighting, and the known benefits of running in the forest, many women still avoid forests due to safety concerns [91]. As a consensus, routes with low traffic, such as residential areas, and open green spaces, such as parks, are viewed as safer for runners.

Injury—Injury could occur from vehicles [10,23,24,35,43,121,122] and interactions with other runners, pedestrians [1,2,15,24,72,111], or cyclists [23,24,35,98]. Congestion from others and traffic cannot only increase injuries but also decrease runners’ overall satisfaction [64]. 

Differing Perceptions—One study found no associations between safety and running but suggests this could be a result of differing methodologies in studies [15]. For example, a questionnaire asking about perceptions of safety may differ from measuring crime rates. Our findings corroborate the study, as similar issues arose, such as non-specified concerns like ‘road safety’ and ‘traffic congestion,’ which could refer to a runner’s fear of injury or to the annoyance of disrupted running flow. Some authors were able to expand their research into hindrances of runnability through qualitative, open-ended methods. For example, some studies discovered that the presence of unleashed dogs was strongly noted as an impediment to running [1,2,98]; yet another study found running with a dog may also entice runners [99]. There is also the concern of locationality; some studies noted a strong negative correlation with runnability and concerns regarding safety from monkeys [21], cougars [24], and bears [23].

### 4.2. Terrain

Terrain refers to the elevation (altitude) and slope (vertical: incline and decline) of the running environment, as well as different surface types. Terrain also covers any specifics of the route length and/or width. 

Runners have different levels of ability and needs, which can be met not only by preferred route aesthetics but also by distance, elevation, slope constraints, and preferred terrain type [38]. Changes in terrain type and gradient force runners to adapt; runners may need to alter their step frequency and length [98], providing additional challenges [24]. Similarly, terrain type may provide extra challenges in different meteorological conditions—a slippery forest floor, for example. In a field study measuring heart rate and speed, it was discovered altitude and gradient increases may have a corresponding effect on heart rate regardless of the route atmosphere (e.g., roads compared to parks) [98]. For habitual runners, ‘terrain’ was ranked higher in importance than ‘convenience’ [67], providing evidence of the characteristics of the environment to be a greater attractant than proximity to home. Runners generally feel comfortable running on trails alone compared to those walking or cycling [37,80] and show a preference for informal trails [65]. 

One study contradicted findings from MapMyFitness and Strava and determined elevation and trails (especially from the park to the boundary) to be the best determiners of park popularity; however, this study reviewed multiple types of physical activity alongside running [71]. There may also be a preference by sex; one study noted males preferred ‘faster’ and ‘harder’ surfaces during Parkrun events, while females preferred ‘trails and grass’ [30]. Additionally, terrain management and preservation of the natural environment are major factors when a runner considers traveling for a running event [94].

#### 4.2.1. Elevation and Slope

Some articles did not differentiate between slope and elevation or mentioned ‘vertical’ as a representative contributing to challenges during analysis. *Elevation* (altitude) can be referred to as ‘elevation gain’ [12,38,104] or ‘vertical gain’ [24] and ‘elevation gradient’ [71] and includes ‘alpine’ [38] and ‘mountains’ [34]. *Slope* (gradient/incline) refers to a rising or falling surface and relates to a ‘running slope’ and/or ‘cross slope’ [57]. Slope can also be referred to as ‘vertical acceleration’ [58], ‘uphill and downhill’ [34,56,102], ‘gradient’ [71,98], ‘hilly’ [38,91], ‘steep’ or ‘steepness’ [38,102,121], ‘hillier’ [30], and ‘hills’ [34]. *Altitude* [34,98,121] and *gradient* [94] were part of some runners’ appeal and thus needed differentiation.

Elevation—Elevation is directly related to running intensity [91,118], and high altitude is negatively correlated with runnability [34,121]. The slope is also directly related to running intensity [39,87,91,118] and running performance [34] and provides a full-body exercise [56]. Both elevation and slope affect runners’ mean heart rates and speed [98] and can be used as an added challenge when there is limited distance for running [91]. Very few studies referred specifically to altitude as height above sea level but evidenced the average runners’ preference for lower altitude [121], perhaps because of the covariate of blue spaces in these terrains [98]. Higher altitude increases heart rate and decreases running speed [98] and is correlated with decreased running activity [34,121]. However, the added challenge of altitude as a feature for events such as SkyRun, mountainous trail running routes held worldwide, is an attractant for running enthusiasts [94]. Additionally, the increase in heart rate at higher altitudes and varied gradients can be used to enhance running performance [98], especially for those training for marathons [34].

Slopes—Environmental runnability is largely affected by variable elements such as seasonality, time of day, and weather [60,121]. Without the possibility of extended running conditions, many runners tend to use slopes to extend their physical activity [91]. Segments that vary in slope attract many runners due to the increased skills required [123] or the runners’ objectives [2]. According to one study, there are three types of runners’ attitudes regarding slopes: (1) as a challenge and to develop strength, especially in compact (e.g., high-density) areas; (2) as an integration into a run, particularly for long-distance runners; or (3) to be avoided completely because they are too challenging or pose a risk of injury [91]. However, as the gradient provides more challenges, runners may encounter fewer obstacles by way of other people. For example, downhill running is an accentuated running strategy for whole-body exercise. Many runners choose to run uphill or downhill as an added challenge because it requires additional techniques and manipulations to a runner’s speed and duration [56].

Slope and Continuity—A negative association of slope (as well as terrain type) with runnability can occur because it may cause conflicts through disruption of running flow or continuity [65], which is one of the most determining factors in runnability [2,10,23,111,124]. There may also be an overrepresentation of elite runners’ routes in popular physical activity apps [39], distorting the attractiveness of steeper running routes to the average runner. Another study mostly discussed proposed guidelines for those with mobility issues (such as participants with visual and mobility impairments); nevertheless, it is important to note that runners have different abilities; running slope and cross slope can be a deterrent to runnability as they affect the level of terrain difficulty [57]. This can also be true for runners with sustained injuries, such as exercise-induced muscle damage (EIMD), a common potential injury during downhill running [56]. 

Trail Runners—For slope enthusiasts, trail running is an off-pavement physical exercise combining running and hiking; many trail runners prefer these softer grounds and buffed-out (a mix of steep and flat sections) routes for the aforementioned additional fitness and strengthening opportunities, additional challenges, and decreased impact on joints [24]. 

#### 4.2.2. Surfaces

Like the lack of differentiation between ‘slope’ and ‘elevation,’ many articles mentioned a preference for running ‘surface’ but did not specify beyond ‘trail’ versus ‘road’ versus ‘sidewalk,’ while others differentiated by material type such as ‘paving,’ ‘asphalt,’ ‘sand,’ ‘stone,’ ‘track,’ ‘terrain,’ etc., making noting surface quality preference difficult. ‘Track,’ for example, was described as “often rough with unpaved surfaces, for mostly agricultural or forestry uses” by one study [35] and as a ‘smooth surface’ in another [43]; additionally, the location of the track was ambiguous as well: ‘jogging track’ [36] or ‘running track in a stadium’ [98]. Others did not specify [34,45,65,120]. Surface type examples found in this study are summarized in Table 8:

Surface Characteristics—Generally, runners prefer wider [35,68,119], well-maintained [23], and smoother [2,35,39,43] surfaces, either off-pavement natural terrain [24] or road [23]. Wider routes reduce conflict among users [71], and smooth surfaces reduce potential injuries [2]. Injuries can also be caused by surface type; one study discussed how runners object to concreting trails as it may cause injuries [59,91,96]. Having a ‘comfortable surface’ is rated highly in many studies [1,12], especially for moderately experienced runners [1]. Some runners showed an increase in preference for synthetic materials [58], while others discussed a preference for leaving the trails to their natural elements, as concreting can ruin the atmosphere [59]. Issues arise when runners prefer informal tracks, which are narrow and typically in high-slope areas; informal tracks have the added technicality but also contribute to environmental degradation [65]. 

Path Type—‘Path type’ is among one of the three most influential characteristics of runnability (other factors were ‘traffic’ and ‘nature’) [38]. Different surfaces can entice or deter runners as they alter difficulty for runners [2]. Comfortable surfaces are an attractant, especially for moderately experienced runners [1]; forests tend to offer the ideal soft ground but may have uneven terrain [91]. Elite or more enthusiastic runners may prefer a more technical surface [2]. For example, during dark and rainy seasons, runners may avoid a dark and slippery forest due to fear of injury, while others may find the slick trail surface and tree-covered routes as an additional challenge [91]. Some studies argue that trails in parks are preferable as they are associated with running intensity [87] and have added challenges from the natural environment (slopes, tree branches, etc.) [94], while others argue that maintained paved surfaces are generally more convenient and reliable (e.g., less likely to slip) during dark and rainy seasons [91]. This may contribute to why some runners view trail running as less competitive than road running [79]. Thermal comfort is important as it helps prevent runners from becoming too cold or hot, which can lead to health issues. Surface temperature is impacted by trail material, which in turn influences runners’ thermal comfort. Unshaded asphalt has high solar absorption and thermal emissivity, causing the trail to be over 10 ° Celsius higher than the air temperature [53]. If the trail has adequate tree canopy coverage, the surface temperature will be reduced through protection from the sun and evapotranspiration [89]. This can be especially problematic for women [112]. Even the direction of the trails has been shown to be significantly effective in mitigating thermal comfort in all seasons except springtime [53]. Trails and public running tracks (regardless of surface type) are used more commonly by those who fear for safety from discrimination [120].

It seems that perhaps while surface type is important, it may not be the most important consideration for a running route—at least in a general sense. One study looking at preferences of running surfaces determined a preference for unpaved or track surfaces but to a small degree [23]. There are also very few studies on non-traditional routes such as cemeteries; runners here noted an attractant being the surface quality of the gravel pathway [66]. 

Importance of Surfaces—There may be other factors affecting a runner’s attraction to a preferred route: asphalt may not be as popular as it is usually confined to roads and therefore has more traffic (people or vehicular), disruption to running flow, running intensity, access to green spaces, and especially connectivity and continuity.

### 4.3. Connectivity of Network and Geometry

Connectivity Defined—Connectivity refers to the continuous nature of a runner’s route; by avoiding stoppage, runners can conserve energy and momentum [2,10]. Rhythm [10] and running flow [2] are important in running as they refer to the desirable or optimal capacity where runners can reach their peak potential, devoid of any interruptions. Connectivity is positively associated with running intensity [87]. 

Connectivity Characteristics—The continuity of the running path has been found to be one of the most important preferences of road runners [23], trail runners [24,87], and unspecified runners [17,39]. Continuity can be encouraged through route length and width [35], trail density [10,17,87], open spaces [35,55], linked segments [12,35,37,43,72], networks connecting green spaces [88], and sometimes including main streets [35]. Further breakdown of the Connectivity of Networks and Geometry include:

(1) Network Connectivity and Interconnected Parks—runners prefer interconnected parks and forests for their running routes, and the length of connected green spaces has a positive effect on mobility [61]. Runners could cover approximately 10 km distance [37], and when comparing trail use by different users, it was determined that runners rarely use unidirectional paths [72]. Runners, therefore, may prefer circular trails [37].
(2) Disruptions of running flow—while traffic-calming infrastructure (e.g., speed bumps, traffic circles) reduces potential injury to runners from vehicles [122], higher-traffic intersections increase the risk of injury and disrupt running flow [10,125]; reduction in runner speed occurs from bottlenecks or angular flows [62], vehicles [10,23,31,35,39], and people (e.g., other trail users) [65]. Street network connectivity can also disrupt running flow when combined with high road density (but has conflicting results as some authors claim it promotes runnability [31,124]), traffic, and traffic accidents [39]. There was no consensus reached on whether primary, secondary, or accessory roads are preferred.
(3) Population Density—The larger determinant of whether the probability of population density was to either be a facilitator or hindrance to runnability was most likely dependent on its disruption of running connectivity and flow; generally, the high-density populated regions inversely reduce jogging flow [23,45]. Pedestrian-Orientated Intersection Density (POID) and population density are linked to increases in physical activity [12,82]. Movement of large crowds can be a disruption to running flow but also hazardous, as it may also result in injury due to stampede [62]. The higher the building density, the more inverse the jogging flow [43]. However, higher population density can also increase the number of runners and, therefore, the runnability of an area [12,20,39,44,82].
(4) High-traffic areas—Areas of high traffic, such as downtown cores, were shown to be a hindrance to running [2,82], often due to safety concerns [10,19,93]. Although some studies determined that running participants increased with higher density [47], there are many covariates to ponder why: there are more people. Therefore, more people may be attracted to running or more opportunities for social engagement on running routes. The neighborhoods promote a feeling of safety away from highways or business districts, or they have little accessibility to open green spaces.

Optimal Connectivity—Forests tend to have the most connectivity as there are fewer disruptions [37,91], except from other trail users [65,72]. Natural environment continuity is associated with interconnected parks and forests [12,43,61], circular trails [37], walking loops [121], footpath networks [31,65,108], and high trail density [87,121], with a preference of up to 10 km in distance [37] and in natural spaces (≥7 ha) [88]. Similarly to trail runners, road runners prefer higher road connectivity [43] and density (with a moderating effect) [43,82]. Other built environment factors positively affecting connectivity include low intersection density [10,23,86], low pedestrian density [2,35,82,111], low traffic areas [2,10,23,82,86], and (generally) being farther from the city center [43,121] (although, again, there were discrepancies in the results of some studies [87]). 

Connectivity Specifics—One study explored the desired speed and turning angle of a bend within complex geometrical settings as they may result in stampede accidents and congestion. The researchers determined an increase in density along the center of the corridor, especially at the bend, which is magnified with higher speeds and would result in a slower pace. Runners would, therefore, prefer to avoid sharp curves [62]. According to another study in running events, satisfaction occurs primarily with running performance, and the most detrimental impact can be caused by congestion, primarily with ‘pinch points’ (e.g., U-turns and right-angle turns) and when paths become narrower. This slows runners down but may also cause bumping into runners and causing potential injury [64]. Supplementary information can be found in Appendix A.

### 4.4. Green Exercise

‘Nature’ is among one of the most mentioned preferences for runners [38]. According to Vujcic et al. (2019), green spaces have three main functions: (1) reducing exposure to pollution (both air and noise), (2) enhancing physical and mental well-being by promoting running through a restorative environment, and (3) facilitating social connections [81]. There are different types of green spaces, and their effects are equally as variable; larger green spaces promote running [96] and better running performance [28], while a lack of green spaces impedes runnability [96]. 

Runners are among the most frequent visitors to green spaces, either alone or with a group [37]. There was an overwhelming consensus that natural attractions are preferred over the built environment, as urban centers may have more interruptions to running flow [119]. Green spaces have been shown to entice and encourage runnability [36,55,88,88,93,96,118,124]. Deelen et al. (2019) discovered that a perceived green and ‘lively’ environment was an attractant for runners of all experiences, a factor more important than internal motivations for running [1]. Its ‘simple’ atmosphere [79], devoid of social constructs, aids in the optimization of a runner’s goals and well-being. Other advantages include technical challenges [100], additive psychological benefits (e.g., stress reduction, greater mental health) when compared to physical activity (PA) indoors, experience and connection to nature [25], and sociability [25,81].

#### 4.4.1. Cemeteries

Increasing urban densification, city expansion, and infrastructure development leave less green space [126]. One study in Malmö, Sweden, studied the perceptions of both runners and non-runners to determine the potential of urban cemeteries as an extension for green exercise. Interestingly, running was observed in all three cemeteries; attractants included the running surface and the proximity to the home as factors [66]. 

Conflicting Views—Approximately 58% of non-runners deemed running in cemeteries to be ‘unacceptable’ or ‘disrespectful.’ While running in cemeteries is not a well-known practice, it may become more commonplace as other green spaces become scarce or as a connection between parks [66].

#### 4.4.2. Dedicated Path

Dedicated Path focuses on the preferred routes of runners with regard to spatial patterns in running behavior. Dedicated paths can be influenced by connectivity but include other factors that influence and promote running in addition to route contiguity: it assesses the importance of a particular path and its users. Dedicated paths can entice experienced runners and influence new runners [67]. Furthermore, increased running distance is correlated with higher self-rated reports of wellness [80].

Path Location and Accessibility—The location of a path and its accessibility are connected to many factors: park popularity [12,71], convenience [67], accessibility through public transit [45,119,121] (although some authors do not agree [124]), proximity [10,17,38,47,72,121] such as distance to home [19,24,26,66,67,76,93,99] or workplace [82], but distance to city center or business areas [43,71,73,87,121] produced inconclusive results. 

Path Characteristics—Beyond connectivity and terrain, running routes have additional factors promoting runnability. The construction and maintenance of paths [11,17,21,37,53,59,61,67,79,91,95,118,121], path width [11,72], and path difficulty [57,72] are factors chosen in a runner’s dedicated path. Furthermore, the formation of pedestrian-oriented (only) spaces [11] is prioritized as they have fewer disruptions from vehicular traffic [2,23]. 

Green Infrastructure (GI) Networks—Green Infrastructure Networks refer to the nodes and links connecting green spaces; GI aids biodiversity, habitats, and connectivity of nature [127]. Accessibility to GI promotes running frequency [121]. One study in Metro Vancouver discovered that over 30% of Strava users’ route segments were continuous with nature [12]. It is not surprising many runners prioritize interconnected GI [12,61] due to the known and perceived advantages of green- and blue spaces. 

Adventure in Running—Many runners prefer adventure on running routes, which can include choosing a remote location or off-trail (or path) routes [71]. This activity creates informal paths [65,68,69] and encourages runners to a particular area. A study combining GPS and a questionnaire noted “scenic view, exploration, and shortcut” to another trail as enticements to a particular park [69]. This was correlated with natural scenery as an importance for runners [17,21,27,32,34,35,43,67,69,96,102,111,121]. 

Additional Correlates—A runner’s dedicated path may also be influenced by a runner’s gender [12,23,24,28,31] and experience [30,67,91], which are beyond the scope of this paper.

#### 4.4.3. Health and Well-Being 

The urban landscape affects physical activity and, therefore, overall health [74]. There is a general consensus in current runnability literature highlighting the connection between green spaces and their features to overall health and well-being [13,40,79]; concurrently, physical activity has been linked to better mental [128] and physical health [129]. Therefore, running in green spaces can enhance each other’s effects [102]. Health and wellness benefits are amplified in green spaces due to their ability to remove environmental stressors such as climate, noise, and air pollution [16,79]. Green spaces support physical activity [76], and one of the main reasons for visiting green spaces is exercise [21]. Those who live in areas with more and better-quality green spaces tend to exercise more and have greater perceived benefits to their overall health [21,81], especially for older individuals who typically have reduced physical activity [76]. Running was a primary physical activity during the SARS-CoV-2 pandemic [20] and increased from pre-pandemic numbers. Green spaces offered social distancing opportunities and a connection with nature while running, limiting the physical and mental strain during this crisis [75]. 

Physical Health—Physical health is a priority for many outdoor runners [79]. One of the primary reasons for green space use is due to physical fitness [21]. Results from this scoping review have determined running in nature has been shown to reduce heart rate [98] and self-reported reduction in stress and headaches [77].

Mental Health—Primarily, mental health effects were reported through green space restorative benefits [21], discussed in further detail in *Spatialities of Running: Meanings and Experiences.* Runners choosing green space reported better self-rated wellness [80] and mindfulness [21], are able to deal better with self-reported nervous issues [81], have a better body self-image [28], have a more positive mood [28], and feel well-balanced [77]. Runners present better self-rated wellness and health when compared to walkers on the same trails [80]. One study noted benefits, particularly for men, who rated better psychological well-being when running in natural spaces [28]. Along with healing effects, green spaces offer a location to promote social networks and their benefits [21,79,81]. 

#### 4.4.4. Parks and Forests 

The runnability scoping review highlighted runners’ preference for a natural environment and identified correlates relating to green spaces. This study discovered a hierarchy in the quality of green spaces in attracting runners [12,21,27,90]; *Green Exercise: Parks and Forests* refers to the characteristics of these larger green spaces and their common amenities. 

Park and Forest Popularity—Previous research on this topic provided evidence to support the known correlates and was able to provide information on useful methodologies to obtain runnability data. Approximately 80% of runners traversed through a park in their route [40], and a high percentage planned their route according to staying in green spaces as long as possible [12]. Parks and forests are associated with many key preferences of runners: clean air [82,93,107], proximity to blue spaces [15,35,86,90,124], shade [47,55,89], visual appeal [27], experiencing nature [25], and creating a meaningful experience during physical activity [60,88,91,103].

Park and Forest Characteristics—Generally, parks can help facilitate a welcoming environment for all runners regardless of age, race, gender, and socioeconomic status [36]. Some studies suggest that *NDVI* (normalized difference vegetation index, top-down greenness) is positively correlated to increased running activity [82], while others suggest the correlation was negative [121] or running intensity was only correlated with *NDVI* in forests and not urban parks [87]. Similarly, *GVI* (green view index) [39,43,85,86] is another visual scape indicator used to determine runner preference for green spaces. Parks and forests generally have an area of unpaved surfaces (e.g., trails) and low path density [2], both preferable to runners. The addition of a jogging track was found to be highly associated with increased activity in a park before and after improvement [36].

Wellness Promotion—Besides physical activity, relaxation and stress reduction are two of the main motivations for people to visit parks [16,26,77]. Regardless of an individual’s reasons and preferences for running, the “ideal run was never indoors” [84]. During the pandemic, running was among the most popular physical activities observed [20], and larger urban green spaces such as parks and forests have been utilized more during times of crisis [75].

Enticing Running—A study by Arifwidodo and Chandrasiri (2021) reviewed park visitors before and after improvements to a jogging track: new developments led to an increase of 17.6% in runners. Improvements considered include park size and separating runners from other physical activities [36]. Impediments to runnability in parks and forests include improper lighting [2,35,47,55,60], air pollution [82,107], and safety concerns [13,55,91].

#### 4.4.5. Green Tourism

*Green Tourism* refers to those who travel to locations for the primary purpose of experiencing a specific running route—primarily trails. It is a growing trend in endurance sports travel [94], including competitive and non-competitive (e.g., Parkrun events [13,30,32]. As of July 2024, 942 events and 2,840 races can be found on the International Trail Running Association (ITRA) website. ITRA represents 165 countries and has posted over 26,000 races since 2013 for approximately 2,500,000 international runners [130]. Popular forms of trail running include mountain running [95] and sky running for elite runners looking for a greater challenge, as well as novice runners and those who prefer running while connecting with nature [94]. This runnability scoping review obtained three studies relating to green tourism: runners’ motivation during SkyRun, a trail event in South Africa [94]; comparing international and Icelandic runners’ attitudes during the 55 km *Laugavegur Ultra Marathon* (LUM) in Iceland [95]; and the non-linear effects of green spaces on active travel [96]. According to ITRA, LUM is less than 20% paved surface [130]. 

Green Tourist Characteristics—Similar to findings for local runners, green tourism participants tend to have higher education [68,80] and income [80,94,96]. Motivations include an interesting but difficult topography, contact with nature, relaxation, and a personal challenge [94,95], and tend not to find exclusivity of the event nor prizes to be a factor [94]. 

Green Tourism Attractants—Both an unfamiliar environment and unique features of the trail are attractants of green tourism, as well as socialization with other tourist runners. Preferences were noted between the international and Icelandic runners: both groups’ primary attraction and motivation were the same—wilderness and personal goals, respectively; however, international runners preferred the ‘wild character’ of the running segment while locals were less interested in the scenic view from the trail and preferred more infrastructure and amenities [95]; these findings were corroborated with Ettema (2015) and Huang et al. (2023b), potentially due to the runners’ familiarity with the landscape. The preferred correlates of green tourism include specific trail characteristics such as surface type and incline, connecting with nature [94], and scenic views of the trail or route [95,96]. 

Goals and Impediments—Motivations include personal goals, challenges, the technicality of the natural environment [94,95], and the importance of being in nature and green spaces [96]. Issues with international trail running include limitations in participants, low revenue, poor marketing and organization [94], and increased environmental degradation similar to the environmental impacts of other sporting events, such as soil damage, trail degradation, and waste production [59,95]. Regardless of the competitiveness of the participant or event, green tourism is reflective of a runner’s dedication as a test against nature requiring time (travel, training) and money (event, accommodations, club memberships, etc.) [94].

#### 4.4.6. Trees and Shade

Trees and Shade have contributed to one of the highest correlates of runnability [23,47]. Trees provide greenness to streetscapes, contributing to road runnability [12,35]. The effects of trees and shade can be seen through both the physiological and mental systems of runners. For example, heart rates tend to be lower near trees [98], and runners noted the positive effects of forests on overall well-being [77]. 

Benefits of Trees—Through observational methods comparing a newly developed urban park to an older forest, the researchers determined runners preferred the shade offered by old-growth trees to open spaces during times of high heat, approximately over 30 °C [89]. While it is true high temperatures affected the number of participants in both the open and forested green spaces, heat and, therefore, seasonality [121] may also affect the results of other studies looking at the attractiveness of environmental runnability. For example, during the evening or extended periods of darkness, runners (especially women) tend to prefer the built environmental features of streetlights and maintained roads for safety [23,91]. Trees become an impediment to runnability when they hinder light and, therefore, safety [91] or the potential of injury from low branches [35]. High canopy density can also lead to unwanted heat retention [53,89]. 

Trees and Health—Trees mitigate meteorological parameters that facilitate microclimate conditions [21,93]: they cool through shading from sunlight and evapotranspiration [89] and affect thermal comfort by slowing down wind speed, increasing relative humidity, and reducing short- and long-wave radiation exposure [53]. Outdoor runners have increased exposure to ultraviolet (UV) light from the sun, causing harmful health conditions such as a higher prevalence of sunburns [110]; combined with higher exertion, sun exposure can lead to an increase in skin cancer markers such as nevi (moles) and lentigines (freckles) [131], increasing the risk of both malignant melanoma and nonmelanoma skin cancer [132].

### 4.5. Blue Spaces

*Blue Spaces* are almost always highly correlated with high runnability [11,12,15,23,25,35,38,39,43,44,55,82,85,86,87,90,99,121,124]; blue spaces are associated with promoting running activity [55], increased time spent and potential of running [12,15,24,35,90,99,124], speed of runners [98], and an increase in running intensity [47,87].

Blue Space Environment—Blue spaces are generally alongside green spaces, which intensifies their preferred characteristics, such as lower pollution [99], generally high socioeconomic regions, and therefore, perceived safer neighborhoods [12] and aesthetics [85]. The most popular park trails were the ones close to water bodies [87]. Among green space design, the specific blue space elements preferred by Europeans are permanent lakes or ponds, followed by streams and rivers, regardless of whether they were natural or restored [35,97].

Blue Space and Health—Regarding physical and mental health, blue spaces have been shown to contribute to better overall well-being [81,99] and a feeling of joy and calm. When comparing runners’ heart rates on different routes, the lowest heart rate was found to be in the park by the sea, followed by the road by the sea [98]. One study reported those living on the coast had reported better general and overall health [99], which can be partially correlated to various forms of outdoor activities such as running; living near blue spaces has been shown to entice physical activity, regardless of whether the spaces were natural or restored [97]. Additionally, the presence of freshwater in neighborhoods has been correlated to better mental health with or without physical activities [99].

### 4.6. Spatialities of Running: Meanings and Experiences

Runners emphasized the importance of connecting with their environment through restorative benefits [1,21,26,77,79,81,102], mental well-being [32], relationships, social needs [99], a sense of community [24,32,75,90], spiritual connections [79], and social traditions [97]. Many of the articles used qualitative analysis to describe runners’ feelings, values, and identities embedded in running beyond mental and physical health. Almost all these experiences were associated with the natural environment and reconnecting with nature in green spaces [60,88,91,102,103] and blue spaces [99]. Ultimately, the atmosphere affects running behavior [90]. 

Exercise as a “Deeply Meaningful Experience”—*Spatialities of Running: Meanings and Experiences* is a complex topic as the interactions between runners and their environment surpass external factors. Both road and trail segments provide a unique experience to each runner, from the physical surroundings to their internal processes [100]. It influences runners’ attitudes and motivations related to perceived characteristics of the environment [1]; for example, running experiences can be enhanced through sensory benefits [32]. Some effects include psychological restoration (e.g., stress reduction and cognitive clarity) [133,134], motivation for physiological benefits (e.g., lower risk for cardiovascular diseases) [135], psychosomatic conditions (e.g., migraines and hypertension) [136], and philosophical experiences [137]. As each of these is unique to everyone [134], *Spatialities of Running: Meanings and Experiences* is divided into six (6) wide and overlapping categories: 

(1) Restorative Effects—Restoration provides a passive effect in mood-enhancing abilities [1,21,26,77,79,81,102]; this is highly affected by a runner’s attitude towards running and their environment. According to Deelen et al. (2019), the runner’s perceived environmental characteristics (how attracted they were to an environment) were more important than motivation or attitude, regardless of running experience, and included a comfortable running surface and a lively green environment [1]. Alternatively, Han (2021) found no such correlation between the environment and physical activity on the emotions or attention of participants, which included more than runners [101]. Furthermore, Han (2017) determined it was the positive effects of nature and not physical activity enhancing runners’ sense of well-being and concluded any exercise would contribute to emotional enhancements (compared to no exercise) [16]. However, the article also stated lower levels of fatigue and nervousness in those who exercised in a natural environment compared to the built environment; these results pose additional questions regarding covariates—this speculation is corroborated by the findings of van den Berg et al. (2019) [134]. Despite obstacles (e.g., pain and time-consuming), many runners note they become immersed in the activity, overwhelming negative feelings about running [41]. 

(2) Enticing Running—New/novice runners have different running experiences than habitual or elite runners, as speed, intensity, experience, and atmosphere will differ [2]. Parkrun is considered a global ‘social movement’ as its popularity has grown due to its focus on encouraging running in a safe and inclusive environment [13,32]. Parkrun hosts non-competitive and community-based [13] mass-participation events, removing much of the stress of becoming a new runner. Its inclusivity encourages health-promotion strategies by removing requirements (e.g., participants can walk to start) with the aim of increasing performance [30]. Its purpose is to motivate and entice participants, regardless of abilities, through a social running event in a natural environment. Inclusivity is crucial to Parkrun events [13]. 

(3) Relationships and Social Connections—Increases in environmental runnability and the probability of running participation were also correlated to social acceptance [13,24,32,75,100] and an enjoyable atmosphere [90]. Physical activity events such as running groups attract frequent runners and entice novice runners [24,32,75,100], especially those with previously low levels of exercise [13,40,60]. Karusisi et al. (2012) determined the social environment to be at least as influential as the physical environment [90], especially with older runners who may not be comfortable alongside younger or experienced runners [89]. A stressful area, or those with little social interaction, are less likely to run [90]. 

(4) Connecting with Green Spaces—Meaningful experiences during physical activity are commonly associated with green spaces [60,88,91,103]. Many runners place meaning in reciprocal relationships with nature [79,133], untouched landscapes [103], and away from the ‘unnaturalness’ of built environments [102]. Trail/off-road running (in nature) is appealing in contrast to the noise and obligations of everyday life [102,133], so much so that one study in Hong Kong discussed the willingness of 95% of green space users to pay for its use, with a high priority on biodiversity conservation [59]. Alternatively, running near cars brings feelings of anger and anxiety [98]. 

(5) ‘Runner’s High’ (Euphoria)—Rochat et al. (2018) reviewed the meanings and experiences of runners during *Tor des Géants*, an ultra-trail marathon in Italy (330 km with 24,000 m of elevation gain), and discussed in detail their phenomenological characteristics (meaningful experiences and interactions during the race), discovering the unique experience during the challenging conditions of these elite events; runners endured trials from the environmental (e.g., difficult terrain), internal processes (e.g., fatigue), and their behavior (e.g., problem-solving how much rest they need) [104]. While safety was a concern among runners, getting lost or having issues with navigation [24] while running was seen as an adventure to others [71], similar to the exploration of those who engage in running tourism [94,102]. 

(6) Mental Well-being—For some, mental well-being includes spiritual connections and reciprocal relationships with nature [79]. In Grabalov’s (2018) study, “54% claimed to hold special feelings when running in cemeteries,” a green space location in a heavily urbanized Malmö [66]. Running is also associated with cultural traditions [97] as it aids mindfulness [21].

### 4.7. Pollution

Environmental Pollution refers to the contamination of the environment with the potential to cause harm to the Earth, its natural resources, and its inhabitants [138]. The runnability scoping review looked at various forms of *Pollution*: *air* (e.g., ground-level ozone (O_3_)), *vehicular* (e.g., traffic-heavy roads), and *noise* (e.g., industrial areas). Only one study found no correlation with air *Pollution* as an impediment to runnability [87]. 

The World Health Organization (WHO) has regarded noise as a form of environmental pollution, noting adverse health effects, including increased stress, feelings of displeasure, and effects on performance [139]. Effects on runners caused by air and vehicular traffic emissions include tiredness, headaches, respiratory diseases, irritation to the eyes and lungs, and cardiac effects [140]. 

The Environmental Protection Agency (EPA) established National Ambient Air Quality Standards (NAAQS) for six criteria air pollutants: carbon monoxide (CO), lead (Pb), nitrogen dioxide (NO_2_), ozone (O_3_), particle pollution (aka particulate matter (PM)), and sulfur dioxide (SO_2_) [141]. The WHO also publishes air quality guidelines according to systematic literature reviews on the same six principal pollutants for global standards [142]. These are amplified in the built environment and contribute to the preference of natural environments for physical activities [143]. While both are significant in decreasing satisfaction during activity, air pollutants tend to have the greatest effect, according to a study from China [144]. For ease of understanding, a summary of common pollutants found in this scoping review (adapted from Bernard et al. (2001) [145]) is summarized in Table 9: 

#### 4.7.1. Air and Traffic Pollution

Air and traffic pollution was found to be one of the strongest impediments to runnability [17,23,82,84,98,111], only secondary to running routes less accessible [35] or less illuminated [10]. Some runners choose to avoid physical activity during times or in regions of poor air quality [46].

Effects of Running on Breathing—During strenuous physical activities such as running, enhanced minute ventilation (V_E_) is required to meet oxygen demands [108,113,115,117]. Runners typically switch to oral breathing, inhale more air to meet oxygen demands, and the increased ventilation rate causes deeper and more frequent breaths [106]. This hyperventilation causes an increased inflow and deposition of pollutants [108], such as black carbon (BC), carbon monoxide (CO), particulate matter (PM_1_, PM_2.5_, PM_10_) concentrations, nitrogen dioxide (NO_2_), ozone (O_3_), and sulfur dioxide (SO_2_) (see Table 9, above). Vehicular exhaust is corroborated to be one of the greatest contributors to air pollution [107,113,115,140], such as common volatile organic compounds (VOCs) in petroleum products [105]. There is a correlation between the increase in the duration of running and a higher level of physical activity, increasing inhalation and, therefore, exposure and dose of pollutants [108,115]. Furthermore, the evaluation of clinical parameters and respiratory tract defense markers demonstrated a significant difference in runners when exposed to pollutants in city streets when compared to a forest [107,108].

Effects of Air Pollution on Health—Some studies quantified the health effects of running in ambient air and traffic pollution. It is estimated there is an increased dose of varied airborne PM five to nine times the normal rate during a marathon, even when the air is relatively clean [117]; 15 min is the approximate maximum for exercise in a moderately polluted environment (when studied alongside extensive meteorological conditions) or when running in a heavily polluted city. After 15 min, the risk to the runner outweighs the benefits of aerobic exercise [33]. If quantifying the volume of air, ~3 h for a runner is equal to approximately 2 days for a sedentary individual breathing the same quality air [113]. Some studies compared varied airborne PM for average versus elite runners and determined a higher pollutant concentration of PM_1_, PM_2.5_, and PM_10_ with average runners, even when the air is relatively clean [112,117], but especially when comparing cities with high and low levels of pollutants [33]. Furthermore, urban settings are associated with increased exposure to pollutants [115]. 

Effects of Air Pollution on Running—A scoping review by Mc Evoy and Buggy (2023) on the respiratory effects of running determined that both CO and PM_2.5_ increase as physical activity increases; most of the evidence determined the effects of pollutants were hazardous to respiratory health (but with mixed results); most importantly, “unanimously concluded that ambient air pollution was associated with slower times in athletes (either just in female athletes or both)” [114]; for example, higher O_3_ concentrations for both sexes [112] and PM_10_ for female runners, potentially because females are more likely to have increased mouth breathing and therefore less likely to trap PM in the nasal cavity [113]. This same article concluded that no other pollutant (CO, O_3_, PM_2.5_, NO_2_, and SO_2_) influenced running performance in men or women (correlated by Marr and Ely (2021)), which was not unanimous with other studies. Only one study found that air quality did not affect the distance or performance of runners regardless of sex but still determined air quality to be a factor in the decision to run outdoors [46]. Qualitative studies noted runners perceive there is a risk when running in pollution [106]; therefore, particular routes may be chosen to avoid pollutants such as pollen, but many runners do not believe air quality affects their performance, although they noted pollutants as an ‘annoyance’ rather than risk unless they had a previous negative experience such as breathlessness [111]. 

Risk of Running in Air Pollution—One study discussed an increased risk of mortality in men related to higher concentrations of particulate matter [117]; while this could not be verified with all PM, a systematic review and analysis specifically researching cardiovascular health and PM_2.5_ found no significant differences in mortality by sex [146]. With that said, cardiovascular and respiratory causes of mortality have been associated with long-term exposure to PM [147]. Countries with high lead concentrations in petrol have resulted in high atmospheric lead pollution seen in blood samples of long-distance runners [109,110]. Running on polluted routes was also associated with higher concentrations of black carbon [116] and NO_2_ (even in low atmospheric levels) [115].

Air Pollution Covariates—Furthermore, all pollutants are amplified by meteorological conditions (temperature, precipitation, humidity, and wind direction and speed) [145], especially lower temperature [113], humidity [114], and intense solar radiation [113,145]. 

#### 4.7.2. Noise Pollution

There are no current studies, at least discovered in this scoping review, that studied *Noise Pollution* as the primary correlate with runnability. However, noise pollution was correlated with the importance of green spaces due to their noise abatement abilities [26,27,81,89,99,101]. Noise pollution was found to be a strong negative association with runnability [23,82,86,111] except when compared to correlates decreasing accessibility [82]. For road runners, avoidance of noise pollution was second only to continuity [23].

Sources of Noise Pollution—Anthropogenic sources are the main cause of noise pollution related to runnability [95,98]. Therefore, noise pollution is most often always associated with the building environment [101] and vehicular traffic [86,98,102]. This is corroborated by a study determining transportation is a main contributor to environmental pollution [148]. Road runners, compared to trail runners, are more likely to encounter noise pollution from traffic [102,111]. High-density residential areas can also contribute to noise pollution from people on nearby roads [27] and crowding [95]. 

Effect of Noise Pollution on Runners—One study reviewed the impact of environmental stressors (including noise pollution) on runners’ fatigue and found no significant differences in the built environment group compared to the nature group. Admittedly, the variance for noise was minimal between the two groups [101] and may not be sufficient. Another study found a positive correlation between running intensity and noise from traffic, but this may be because higher population density regions have more facilities that promote active travel [96], which may attract more runners, especially in higher Socioeconomic Status (SES) neighborhoods [20,90,118,119], and generally contain higher traffic [82,86], which correlates with other findings suggesting runners tend to be of higher income economies [20,59,80,82,94]. In contrast, many other studies found natural settings, such as green spaces and blue spaces, are preferred by runners due to their capacity to buffer noise [27,81,89,99]. Noise pollution has been linked to detrimental health effects such as anger, depression, and cognitive impairment [101,149,150]. Municipalities with high noise pollution may be susceptible to lower longevity [21] and tend to have higher cases of nervous health disorders [81].

### 4.8. Socioeconomic Status and Marginalized Groups

Runnability of a neighborhood is largely determined by a runner’s perceived pleasantness of routes [86,90] defined by community development [45] and aesthetics [87,119] typically associated with higher SES areas [20,90,118,119]. Running is almost 50% less likely to occur in low (compared to moderate) SES neighborhoods [12].

SES, Environment, and Residents—Findings overwhelmingly pointed to green space as a prime correlate of runnability, but the results of one study indicate that the SES of the neighborhood may be more influential than the presence of green space [118]; however, this may be due to additional factors: primarily, running routes connecting parks most likely occurred in SES neighborhoods [40,45] with higher housing prices [86] and residents with higher education [15,17,25,31,37,69,80,88,90,94,99], but this also resulted from those in a low SES neighborhood being less attracted to running [99,118]. There may also be factors that contribute to ongoing exclusion from green spaces, such as limited accessibility to parks in lower SES communities [13,26,45] and ongoing neighborhood conflict [37]; known high-crime locations are a high deterrent to runnability, especially for women [19]. Indeed, one of the few studies that did not link the addition of green spaces to an increase in physical activity was when a greenway was constructed in an already disadvantaged community with a high crime rate [118]. The aesthetics and safety of high SES neighborhoods would also correlate to why their residents tend to be more attracted to running [99,118]. 

Running and Population Density—There is no consensus on the effect of population density on runnability thus far. It appears there is a moderating effect on density; runners prefer more solidarity from high population traffic, but highly confined streets, especially with little illumination, are a hindrance [47]. Ultimately, should runners choose the built environment over the natural environment, the perfect runnable environment in terms of housing and population density refers to low-density but high-priced homes [86]. The larger determinant of whether the probability of population density was to either be a facilitator or hindrance to runnability was most likely dependent on its disruption of running connectivity and flow. Running (or bicycling, as it was not differentiated) only rose from 4% to 9% after the construction of a 1.5-mile urban greenway in a disadvantaged and high-crime community, but findings were similar at a comparable site [118]. Another study found no correlation between runners’ preferences and an association between neighborhood SES, but this may be a result of different methodologies (e.g., questionnaires about perceptions versus reviewing income, for example) [15]. Runnability hindrances occur due to barriers. 

Running and Discrimination—Racial discrimination and harassment can cause further physical and mental stress [151] and can also hinder runnability. A study comparing runners of different racial backgrounds discovered social inequality and racism to be a significant barrier to outdoor physical activity. Outdoor spaces increase stress due to discrimination, amplified when the African American runners were of higher education and income and/or living in a higher SES, predominantly white, neighborhoods. Furthermore, the author(s) speculate those who fear for their safety due to racism or prejudice may prefer runner-specific venues such as trails or tracks [120]. This limitation to runnability can contribute to why people of color have higher and more complicated medical needs, such as cardiovascular diseases [152]. 

Running and Gender—A study reviewing the characteristics of parkrun events with the aim of overcoming participation barriers and increasing inclusivity noted a gender gap in physical activity in the United Kingdom [30]. One study discovered most women (as high as 84%) and over half of men in London have experienced physical or verbal harassment or concerns about safety while running. These barriers contribute to ongoing runnability hindrances; many runners find their running route location or times are, therefore, limited [54]. Generally, women feel more comfortable in urban parks compared to other outdoor spaces while running [74], typically for safety [37].

### 4.9. Additional Correlates

As highlighted in a majority of runnability studies, green spaces are positively correlated. Many authors discussed levels of greenness; however, this study did not differentiate between eye-level streetscapes/greenness/greenery [85,96] and subjective streetscapes [119], eye-level greenness versus top–down greenness, nor did we differentiate between types of green spaces with Normalized Difference Vegetation Index (NDVI) and the eye-level greenness by Green View Index (GVI) [85]. Additionally, we did not account for land use mix type, as its variability (moderate or high, for example) [2,39,85,86,87,96,102,119] can be ambiguous and may produce different results. According to a recent study reviewing the relationship between green space exposure and mental health, only eye-level exposure is significant in reducing stress, and there was no significant association when comparing street- and top–down NDVI-based exposure [153]. 

Furthermore, we did not differentiate between street environment versus built environment, as has already been completed by Dong et al. (2023) [119], and macro-scale versus micro-scale built environment features, as was already completed by Jiang et al. (2022) [63]. 

Finally, there are debatable correlates or detriments to runnability. These include urbanicity, population density, and distance to home and/or workplace. These findings are thus far inconsistent and were therefore not included in the runnability scoping review.

### 4.10. Limitations 

The primary focus of this scoping review was to determine generalizable runnability correlates. Our researchers determined the large volume of current runnability research contained many similar correlates but with debatable results; articles found in this runnability scoping review were subject to researcher bias. This paper only included correlates discovered in the scoping review process as defined by Arksey and O’Malley (2005). Limitations of scoping reviews include difficulty regarding the breadth of study (e.g., number of correlates) and less legitimate articles, as scoping reviews do not appraise the quality of evidence [48]. There is a possibility of missing correlates or undiscovered covariate influences either through the search process or unintentional omission; furthermore, limitations in current research or findings not unanimously agreed upon present additional challenges. Within the current runnability research, some correlates were not generalizable, or qualitative data, while important to determine runner experience, cannot be related to multiple studies. Examples include abstract concepts (e.g., ‘pleasantness’ and visual quality), unique characteristics in regions or cultural differences for segment preference, and axes of difference (e.g., gender, disabilities). Research completed during SARS-CoV-2 or with data from the pandemic timeframe or with COVID-19 as a correlate [20,38,75,87,97,124] may not provide generalizable data outside an aerosol-transmitted pandemic where runners may have taken extra precautions. Another difficulty resulted from not exclusively researching runnability; some studies viewed PA but did not explicitly differentiate correlates to running (e.g., Hu and Zhao (2022) discussed outdoor recreation, which included “sitting and barbecue” [55]). On the opposite end of the spectrum, some articles in this scoping review contained many correlates, making it challenging to determine their individual influence on runnability, especially when categorization was not apparent (e.g., a study explicitly studying the effects of pollution on running versus a study reviewing pollution as a potential correlate alongside other green space effects). 

## 5. Conclusions

The runnability scoping review reviewed all relevant literature, which used a variety of methodologies, including GPS tracking and GIS analysis, observations, surveys, interviews, or a combination thereof on the preferences of runners. An extensive analysis provided the correlates for the optimum running experience. Overall, green spaces are preferred to the built environment for all runners. Runners tend to choose larger parks or navigate to multiple parks on their routes. Green spaces are correlated to many other facilitators of runnability: high connectivity, blue spaces, low noise and air pollution, high continuity, and optimal connectivity. 

The most contentious correlates relate to slope and surface. The slope is sometimes sought out as an added strengthening challenge and used as an alternative to distance running. The characteristics of the surface were rated highly important, but there is no consensus on the optimum surface for all runners: road versus trail runners or smooth versus uneven preferences are dependent on the individual, their experience, and even seasonality. 

The Model Running Route—The average runner prioritizes safety. Ideally, the average runner prefers a route with ample connectivity, such as a continuous trail at low elevation, a soft and comfortable running surface with gentle slopes within a moderately tree-covered green space, and alongside a blue space. The route should facilitate running flow, such as a low-density pedestrian-only route away from vehicles and other forms of air and noise pollution. Typically, these routes are found in higher SES neighborhoods, which may contribute to most runners having higher wealth and/or education. 

The Elite Running Route—Experienced runners prioritize terrain and connectivity. They tend to seek out additional challenges for whole-body workouts or training purposes. Formal and informal trails provide softer ground with buffed-out paths; whether purely uphill or down, or varied running and/or cross slopes, gradient amplifies route difficulty and running intensity. The most extreme runners may also choose routes in high-altitude regions, informal or unfamiliar trails, and travel for running events. 

Results determined there are significant deterrents to runnability. Lower SES neighborhoods are associated with ongoing neighborhood conflict and crime, contributing to both real and perceived safety concerns. They also tend to lack many of the runnability correlates attracting runners to a location: accessibility to parks, green and/or blue spaces, and amenities such as sidewalks. Furthermore, vulnerable populations are met with many runnability barriers, including harassment of women and discrimination against people of color. Regions with high levels of air pollutants, such as industrialized areas or high-traffic locations, significantly decrease runnability. Vehicles and vehicular traffic were determined to be one of the strongest impediments as they contribute to many other runnability deterrents: disruption of flow, excessive noise, probability of injury, and pollution. Generally, traffic decreases running satisfaction.

Future Research—Research in the future should assess debated runnability correlates and covariates such as accessibility through public transit [35,43,45,119,124], distance to home [19,24,26,66,67,76,82,93,99] or workplace [82,108], and/or distance to city center or business areas [43,71,73,87,121], amenities (e.g., washrooms, drinking fountains, benches, trash disposal) [11,21,23,36,37,40,43,59,69,79,85,89,98,99,103,119,121], population density [12,15,31,35,39,43,44,45,72,82,85,86,87,88,119,121], and should include runner experience (amateur versus elite) [1,2,10,30,35,47,64,67,94,98,107,112,114,116,117], gender and/or age [12,15,17,21,23,24,24,28,28,31,37,59,69,70,75,79,82,85,86,93,102,111,112,114,116] as correlates may differ among populations.

## Figures and Tables

**Table 7:** Summary of Runnability Themes.

Themes		Examples	Articles Discussion Theme as Main Argument
SAFETY		Injury, harassment, conflict reduction, daylight, streetlights, animal/people attacks, seasonality	Brockschmidt and Wadey (2022) [54], Hu and Zhao (2022) [55]
TERRAIN	Elevation and Slope	Vertical acceleration, uphill/downhill running, elevation	Bontemps et al. (2020) [56], Longmuir et al. (2003) [57], Ren et al. (2020) [34].
	Surfaces	Trail (e.g., woodchip), road, concrete, synthetic running track, etc.	Boey et al. (2017) [58], Ribet and Brander (2020) [59], Rosenkrantz et al. (2024) [24], Thuany et al. (2023) [60]
CONNECTIVITY OF NETWORK and GEOMETRY		Facilitators and disruptors of running flow, distance to preferred running location, accessibility, route conflicts	Cai et al. (2023) [61], Farías-Torbidoni et al. (2023) [37], Hannun et al. (2022) [62], Jiang et al. (2022) [63], Liu et al. (2023) [43], Peckover et al. (2022) [64], Santos et al. (2016) [65], Schuurman et al. (2021) [23], Shashank et al. (2022) [10], Zhong et al. (2022) [31]
GREEN EXERCISE	Cemeteries	Routes that include a cemetery	Grabalov (2018) [66]
	Dedicated Path	Spatial patterns and running behaviors	Gordon et al. (2004) [67], Harden et al. (2022) [12], Harden et al. (2024) [11], Korpilo et al. (2017) [68], Korpilo et al. (2018) [69], Liu et al. (2022) [70], Norman and Pickering (2019) [71], Norman et al. (2019) [72], Sharpe et al. (2004) [17], Shreepriya et al. (2021) [38], Suminski et al. (2008) [73]
	Health and Well-being	Includes physical health (cardiac conditions, diabetes, headaches, etc.) and mental well-being (stress reduction, depression, etc.)	Barnfield (2016) [74], Bherwani et al. (2021) [75], Campos-Uscanga et al. (2022) [28], Dalton et al. (2016) [76], Fischer and Gopal (2021) [20], Hansmann et al. (2007) [77], Hobin et al. (2020) [78], MacBride-Stewart (2019) [79], Nath et al. (2018) [21], Smiley et al. (2020) [80], Vujcic et al. (2019) [81]
	Parks and Forests	Park and forest-based physical activity. Includes all green spaces regardless of size	Adlakha et al. (2014) [40], Arifwidodo and Chandrasiri (2021) [36], Boakye et al. (2021) [82], Calogiuri and Elliott (2017) [25], Deelen et al. (2017) [15], Florgård and Forsberg (2006) [83], Grigoletto et al. (2022) [26], Hitchings and Latham (2016) [84], Huang et al. (2022) [85], Huang et al. (2023) [39], Huang et al. (2023) [86], Huang et al. (2023) [87], , Jansen et al. (2017) [88], Kabisch and Kraemer (2020) [89], Karusisi et al. (2012) [90], Kothencz et al. (2017) [27], Lepoša et al. (2023) [91], Yang et al. (2023) [92], Yang et al. (2024) [44], Yildirim et al. (2020) [93]
	Tourism	Runners traveling to locations specifically for running atmosphere	Myburgh and Kruger (2021) [94], Ólafsdóttir et al. (2021) [95], Yang et al. (2024) [96]
	Trees and Shade	Protection against natural elements such as sun and wind	Alawadhi (2022) [53]
BLUE SPACES		Natural or man-made lakes, seas, rivers, streams, etc.	Jakstis et al. (2023) [97], Paraskevopoulou et al. (2022) [98], Pasanen et al. (2019) [99], Zhang et al. (2023) [47]
SPATIALITIES OF RUNNING: MEANINGS AND EXPERIENCES		Emotional wellness, connection to nature and others through social running, clubs, or mass participation events	Chambers and Poidomani (2022) [100], Cook et al. (2016) [41], de Vries et al. (2022) [13], Deelen et al. (2019) [1], Ettema (2016) [2], Han (2017) [16], Han (2021) [101], MacBride-Stewart (2019) [102], O’Brien (2019) [32], Qviström (2016) [103], Rochat et al. (2018) [104]
POLLUTION	Air and Traffic	Pollution from traffic and other vehicular congestion, low air qualities	Blair et al. (2010) [105], Chow and Chen (2022) [106], Cavalcante de Sá et al. (2016) [107], Dirks et al. (2012) [108], Grobler et al. (1986) [109], Grobler et al. (1992) [110], Hodgson and Hitchings (2018) [111], Hodgson et al. (2022) [112], Hu et al. (2017) [46], Marr and Ely (2010) [113], Mc Evoy and Buggy (2023) [114], Pasqua et al. (2018) [33], Perdelli et al. (2000) [115], Pun and Ho (2019) [116], Zoladz and Nieckarz (2021) [117]
	Noise	Noise disturbances	n/a
SES AND MARGINALIZED GROUPS		Disadvantaged urban communities, low versus high SES neighborhoods, unequal access to green spaces	Auchincloss et al. (2019) [118], Dias et al. (2022) [19], Dong et al. (2023) [119], Gilburn (2023) [30], Hornbuckle (2021) [120], Song and Zhang (2021) [45]

**Table 8:** Surface Type Examples.

Group	Subgroups and Considerations	Article Examples
Grass	natural grass	Ettema (2016) [2], Boey et al. (2017) [58], Jiang et al. (2022) [63]
Gravel	Small rocks, non-synthetic, non-paved	Grabalov (2018) [66]
Informal	narrow, not intended for use	Santos et al. (2016) [65]
Paved	asphalt, concrete, smooth surfaces may be a sidewalk or trail	Ettema (2016) [2], Huang et al. (2023) [39], Boey et al. (2017) [58], Ribet and Brander (2020) [59], Jiang et al. (2022) [63], MacBride-Stewart (2019) [102]
Road/streets	smooth surface presence of vehicles	Schuurman et al. (2021) [23], Ren et al. (2020) [34], Jiang et al. (2022) [63], Dong et al. (2023) [119]
Sidewalk	usually paved, near streets	Schuurman et al. (2021) [23], Ren et al. (2020) [34], Song and Zhang (2021) [45], Thuany et al. (2023) [60], Jiang et al. (2022) [63], Liu et al. (2022) [70], Dong et al. (2023) [119], Hornbuckle (2021) [120]
Synthetic	turf, rubber-modified concrete, rubber tracks	Liu et al. (2023) [43], Boey et al. (2017) [58], Liu et al. (2022) [70], Yildirim et al. (2020) [93]
Trail	forest and/or park paved, woodchip, natural	Ettema (2016) [2], Rosenkrantz et al. (2024) [24], O’Brien (2019) [32], Farías-Torbidoni et al. (2023) [37], Lepoša et al. (2023) [91]
Uneven	non-smooth pavement, muddy, holes	Ettema (2016) [2], Jiang et al. (2022) [63], MacBride-Stewart (2019) [102].
Unspecified	‘walking loops’, ‘footpaths’	Zhong et al. (2022) [31], Liu et al. (2023) [43], Santos et al. (2016) [65], Liu et al. (2022) [70], Dirks et al. (2012) [108]

**Table 9:** Common Air Pollutants.

Chemical Symbol	Name	Article(s)
BC	black carbon	Pun and Ho (2019) [116]
COx	unspecified carbon oxides	Mc Evoy and Buggy (2023) [114]
CO	carbon monoxide	Dirks et al. (2012) [108], Marr and Ely (2010) [113]
O_3_	(ground-level) ozone	Cavalcante de Sá et al. (2016) [107], Hodgson et al. (2022) [112], Marr and Ely (2010) [113], Mc Evoy and Buggy (2023) [114], Pun and Ho (2019) [116]
PM_1_	≤1 μm particulate matter (especially dangerous due to small size)	Zoladz and Nieckarz (2021) [117]
PM_2.5_	≤2.5 μm particulate matter (e.g., vehicle exhaust, wildfire smoke)	Cavalcante de Sá et al. (2016) [107], Hodgson et al. (2022) [112], Marr and Ely (2010) [113], Mc Evoy and Buggy (2023) [114], Pasqua et al. (2018) [33], Zoladz and Nieckarz (2021) [117]
PM_10_	≤10 μm particulate matter (e.g., mold spores, bacteria, airborne viruses)	Marr and Ely (2010) [113], Mc Evoy and Buggy (2023) [114], Pasqua et al. (2018) [33], Zoladz and Nieckarz (2021) [117]
NO_x_	unspecified nitrogen oxides	Mc Evoy and Buggy (2023) [114]
NO_2_	nitrogen dioxide	Cavalcante de Sá et al. (2016) [107], Hodgson et al. (2022) [112], Marr and Ely (2010) [113], Perdelli et al. (2000) [115]
SOx	unspecified sulfur oxides	Mc Evoy and Buggy (2023) [114]
SO_2_	sulfur dioxide	Marr and Ely (2010) [113]

## Data Availability

The data presented in this study were obtained through searches of publicly available library databases.

## Appendix A

Section 4.3 Connectivity of Network and Geometry

Connectivity was determined to be one of the highest correlates to runnability. The following papers provide an in-depth analysis of specifics related to connectivity, geometry, or both:

(1) See Schuurman et al. (2021) for road runner preferences with a high focus on running flow disruptions due to traffic [23].

(2) See Jiang et al. (2022) for a further breakdown of the preferences of road runners related to connectivity: trunk, primary, secondary, and tertiary roads are generally wider and connect to other major streets, lessening disruption to runner flow by congestion, and have higher accessibility and continuity [35].

(3) See Hannun et al. (2022) for information on spatial density distributions and spatial distribution of speed to see how runners’ flow is affected by geometrical layouts [62].

Section 4.4.3 Health and Well-Being

The scoping review captured a research proposal by Hobin et al. (2020), a plan to determine if urban trail network expansion will reduce cardiovascular disease in Winnipeg [78]. Its results will provide additional evidence to support the role of green spaces in health promotion.

Section 4.7.1 Air and Traffic Pollution

Mc Evoy and Buggy (2023) [114] provided an exemplary article on the effects of ambient air pollution on runners beyond the scope of this paper; its summary includes all articles found in the *Air and Traffic Pollution* section of the runnability scoping review apart from Perdelli et al. (2000) [115].
